# Multi-omics integrative analysis reveals novel genetic loci and candidate genes for ischemic stroke

**DOI:** 10.1016/j.omtn.2025.102633

**Published:** 2025-07-17

**Authors:** Min Wang, Chong Xu, Xiaoshan Du, Tian Zhu, Xitong Yang, Fuhui Duan, Guangyan Wang, Yongchun Zuo, Huaqiu Chen, Guangming Wang

**Affiliations:** 1 School of Clinical Medicine, Dali University, Dali, Yunnan 671000, China; 2 Department of Geriatrics, South District of Hefei First People’s Hospital, Hefei, Anhui Province 230000, China; 3 Center of Genetic Testing, The First Affiliated Hospital of Dali University, Dali, Yunnan Province 671000, China; 4 Department of Clinical Laboratory, Chuxiong Yi Autonomous Prefecture People’s Hospital, Chuxiong 675000, China; 5 State Key Laboratory of Reproductive Regulation and Breeding of Grassland Livestock, Institutes of Biomedical Sciences, College of Life Sciences, Inner Mongolia University, Hohhot 010021, China; 6 Department of Laboratory, Xichang People’s Hospital, Xichang, Sichuan 615000, China

**Keywords:** MT: Bioinformatics, ischemic stroke, IS, genetic loci, Mendelian randomization, LLM-based knowledge base

## Abstract

Ischemic stroke (IS) is a major cause of disability and mortality, but its genetic basis remains poorly understood. This study integrates data from three large-scale genome-wide association studies (GWASs), the GWAS Catalog, MEGASTROKE, and Open GWAS, to identify novel genetic loci linked to IS. Our meta-analysis revealed 124 new IS-associated loci, with enrichment in genes involved in cerebrovascular function, inflammation, and metabolism. Candidate genes like *CPNE1*, *HSD17B12*, and *SFXN4* are linked to lipid metabolism, immune response, and iron metabolism, indicating diverse pathogenic mechanisms in IS. Further analyses, including expression quantitative trait locus (eQTL) and protein quantitative trait locus (pQTL), confirmed the relevance of these genes in the brain. Mendelian randomization and colocalization analyses highlighted seven genes with potential causal relationships to IS. Single-cell RNA sequencing identified differential gene expression in endothelial cells, implicating these genes in vascular dysfunction. Functional validation in knockout mouse models showed HSD17B12’s role in fatty acid metabolism, linking it to cerebrovascular diseases. We also developed StrokeGene, an intelligent assistant based on large language models (LLMs) to aid IS genetic research. StrokeGene offers insights into IS pathophysiology. Collectively, these findings substantially advance the understanding of IS genetics and provide a foundation for precision medicine strategies in stroke prevention and treatment.

## Graphical abstract

**Figure:**
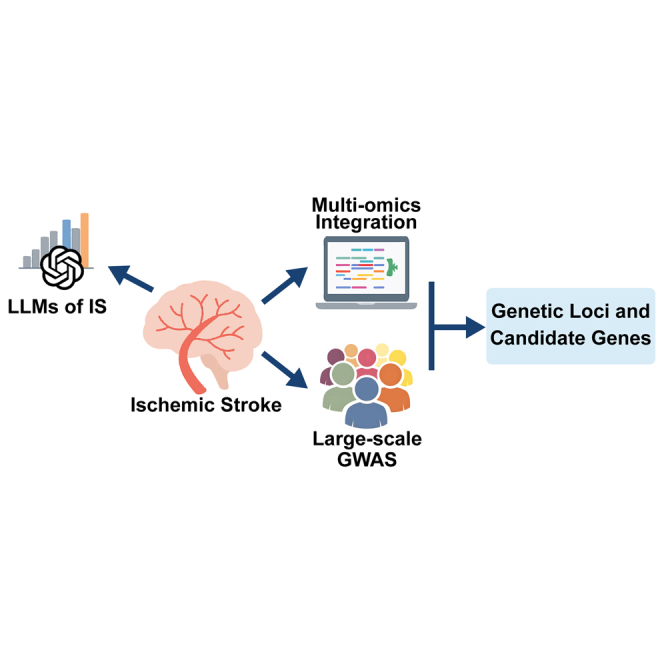

## Teaser

Multi-omics integration of large-scale GWASs uncovers novel genetic loci and candidate genes underlying ischemic stroke risk. These findings reveal key roles for vascular, immune, and metabolic pathways and introduce an AI-assisted framework for prioritizing causal genes, advancing mechanistic insight and translational potential in stroke genetics.

## Introduction

Ischemic stroke (IS) remains one of the leading causes of mortality and long-term disability worldwide, with a steadily increasing incidence, particularly among aging populations.^1^^,^^2^ Pathologically, IS primarily results from cerebral vessel occlusion, causing ischemia, hypoxia, neuronal damage, and subsequent functional deficits.^2^ Although significant progress has been achieved in acute stroke therapies such as intravenous thrombolysis and mechanical thrombectomy, many patients continue to face severe long-term impairments, including motor dysfunction, speech deficits, and cognitive decline.^3^^,^^4^ Therefore, beyond acute intervention, a deeper understanding of the molecular mechanisms underlying IS is essential to develop targeted prevention strategies and therapeutic approaches aimed at reducing incidence and recurrence rates.^5^^,^^6^

Recent advances in stroke pathophysiology highlight its complexity, involving multiple pathological factors such as vascular dysfunction, metabolic disturbances, and neuroinflammatory responses.^7^^,^^8^ Among these processes, endothelial injury, coagulation abnormalities, blood-brain barrier disruption, and immune activation are particularly significant in driving IS pathology.^9^^,^^10^ At the cellular level, oxidative stress, apoptosis, and mitochondrial dysfunction further amplify neuronal injury.^11^ However, despite these insights, the precise molecular mechanisms underlying IS remain elusive due to the intricate interplay between genetic susceptibility and environmental risk factors.^12^^,^^13^ Exploring the genetic architecture of IS is thus critical for identifying potential therapeutic targets and refining precision medicine approaches.^14^^,^^15^^,^^16^^,^^17^

Genome-wide association studies (GWASs) have significantly enhanced our understanding of IS genetics by identifying numerous susceptibility loci associated with cerebrovascular diseases.^18^^,^^19^ Large-scale efforts, including the MEGASTROKE consortium and the GWAS Catalog, have provided extensive candidate genetic loci linked to stroke risk, laying the groundwork for functional and mechanistic studies.^18^^,^^20^ Nevertheless, while GWAS have effectively pinpointed multiple genetic variants associated with IS, the functional implications of these variants and their precise roles in disease pathogenesis remain inadequately explored.^21^ Moreover, existing genetic studies have largely focused on identifying individual genetic markers without comprehensive functional characterization or integration with multi-omics data.^21^^,^^22^^,^^23^^,^^24^ Combining GWAS findings with transcriptomic and proteomic datasets can thus offer deeper insights, helping to reveal novel causative genes and elucidate their biological roles in IS.

**Figure 1:**
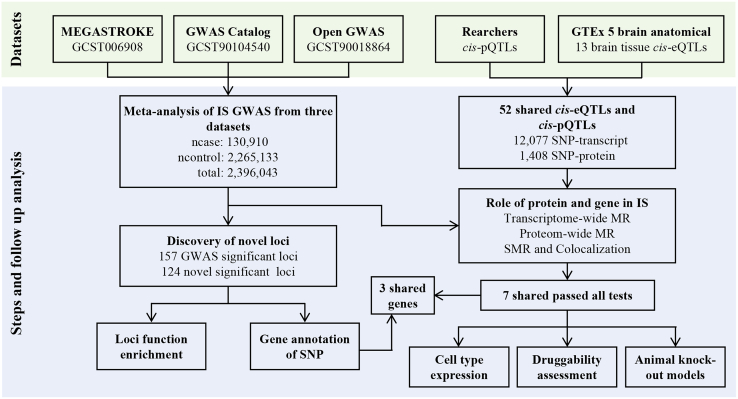
Workflow of dataset and candidate gene analyses IS, ischemic stroke; GWAS, genome-wide association study; SNP, single-nucleotide polymorphism; eQTL, expression quantitative trait loci; pQTL, protein quantitative trait loci; MR, mendelian randomization; SMR, summary-data-based Mendelian randomization.

In the present study, we performed a systematic, integrative analysis of IS-associated genetic loci using three extensive GWAS datasets: GWAS Catalog,^25^ MEGASTROKE,^18^ and Open GWAS.^26^ Our analytical pipeline involved stringent genetic variant screening, sophisticated gene annotation utilizing advanced methodologies such as polygenic priority score (PoPS), and comprehensive integration of multi-omics data encompassing expression quantitative trait loci (eQTLs) and protein quantitative trait loci (pQTLs). Additionally, through Mendelian randomization (MR) and colocalization analyses, we identified novel genetic loci and prioritized candidate genes potentially involved in IS pathogenesis (Figure 1). Furthermore, we developed StrokeGene, an intelligent research assistant based on large language models (LLMs), designed to facilitate in-depth exploration and interpretation of IS genetics. This multilayered integrative approach is anticipated to yield novel insights into the genetic basis of IS and to identify promising therapeutic targets.

## Results

### Genome-wide meta-analysis identifies 124 novel loci associated with IS

**Figure 2:**
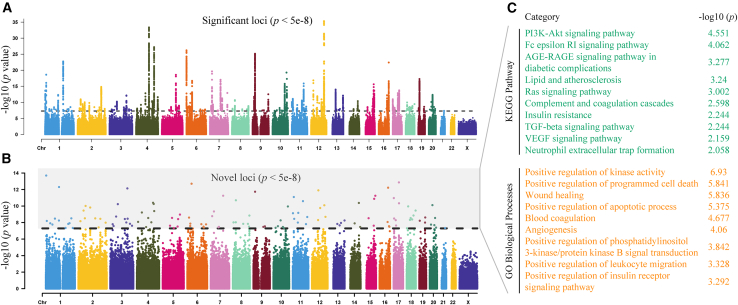
Manhattan plots showing associations with IS from a GWAS meta-analysis (A) Manhattan plot showing the *p* value of association for each loci from GWAS meta-analysis. The *x* axis represents chromosomal location, and the *y* axis represents −log10 (*p* value). The black dotted line corresponds to the genome-wide significance threshold (*p* < 5 × 10^−8^). (B) Manhattan plot showing the −log10 (*p* value) of association for new loci plotted against genomic position on the *x* axis. The black dotted line corresponds to a *p* threshold of 5 × 10^−8^. (C) The KEGG pathway (top) and GO biological process (bottom) analyses of the gene annotation of new loci. KEGG, Kyoto Encyclopedia of Genes and Genomes; GO, Gene Ontology.

We conducted a comprehensive meta-analysis of GWAS results from three major IS datasets: GWAS Catalog (GCST90104540; 62,100 cases and 1,234,808 controls), MEGASTROKE (34,593 cases and 624,214 controls), and Open GWAS (ebi-a-GCST006908; 34,217 cases and 406,111 controls). Following rigorous quality control and standardization procedures, our analysis encompassed a total of 24,640,797 genetic variants. Of these, 157 variants reached genome-wide significance (*p* < 5 × 10^−8^; Figure 2A; Figure S1). Specifically, 23 significant single-nucleotide polymorphisms (SNPs) originated from the GWAS Catalog dataset, 15 from MEGASTROKE, and 9 from Open GWAS, with 33 SNPs overlapping across datasets. Critically, 124 of these 157 variants represented novel genetic loci for IS, defined as variants situated over 500 kb from previously documented loci (Figure 2B).

To functionally interpret these novel genetic findings, we annotated candidate genes located within ±500 kb of each index SNP. We employed a dual annotation strategy, combining the nearest-gene method with the PoPS approach, the latter integrating multi-dimensional genomic features for enhanced functional prediction accuracy (Table 1; Table S1). Subsequent functional enrichment analyses, including Gene Ontology (GO) and Kyoto Encyclopedia of Genes and Genomes (KEGG) pathways, revealed significant associations with key biological pathways implicated in IS pathogenesis, notably the “AGE-RAGE signaling pathway in diabetic complications,” “ipid metabolism and atherosclerosis,” as well as cellular processes such as “positive regulation of apoptotic signaling”and “eukocyte migration” (Figure 2C; Table S2).

**Table 1:** Loci reported for IS in the meta-analysis of three IS GWAS datasets

SNP	Chr	Pos	Nearest gene	PoPS gene	Allele1	Allele2	Eaf	Beta	StdErr	*p* value	Direction
**Novel variants**											

rs4507142	2	44086591	***ABCG5***	***ABCG5***	a	g	0.9344	−0.0696	0.0111	3.53E−10	–
rs113802622	15	86276596	***AKAP13***	***AKAP13***	a	c	0.9613	−0.0989	0.0146	1.34E−11	–
rs112419397	2	144331595	***ARHGAP15***	***ARHGAP15***	t	c	0.9417	0.0706	0.0119	3.13E−09	+++
rs10938202	4	42652337	***ATP8A1***	***ATP8A1***	t	c	0.9335	0.0661	0.0107	6.05E−10	+++
rs4718964	7	70038969	***AUTS2***	***AUTS2***	t	g	0.5613	0.027	0.0049	3.31E−08	+++
rs76675188	12	105725660	***C12orf75***	***C12orf75***	a	g	0.9404	−0.0763	0.0117	8.05E−11	–
rs10828606	10	18665710	***CACNB2***	***CACNB2***	a	g	0.7235	−0.0301	0.0055	4.30E−08	–
rs6024418	20	54369866	***CBLN4***	***CBLN4***	t	g	0.6995	0.0331	0.006	4.08E−08	+++
rs73002403	18	58793768	***CDH20***	***CDH20***	t	g	0.9753	0.1054	0.0174	1.40E−09	+++
rs3176336	6	36648816	***CDKN1A***	***CDKN1A***	a	t	0.5758	0.037	0.005	1.97E−13	+++
rs9564877	13	72683932	***DACH1***	***DACH1***	a	g	0.8621	−0.0437	0.0078	2.19E−08	–
rs4298492	8	26024889	***EBF2***	***EBF2***	a	g	0.7522	0.0372	0.0056	2.00E−11	+++
rs702757	4	148409272	***EDNRA***	***EDNRA***	a	t	0.702	0.0328	0.0053	4.07E−10	+++
rs6577837	8	138801988	***FAM135B***	***FAM135B***	a	g	0.5804	−0.034	0.0061	3.09E−08	–
rs1006856	14	92353962	***FBLN5***	***FBLN5***	c	g	0.605	0.0322	0.0049	4.22E−11	+++
rs72889922	2	173321704	***ITGA6***	***ITGA6***	a	g	0.9855	0.1748	0.0318	3.85E−08	+++
rs6438857	3	124557643	***ITGB5***	***ITGB5***	t	c	0.5495	0.0282	0.0048	3.72E−09	+++
rs6074239	20	11056468	***JAG1***	***JAG1***	t	c	0.895	−0.0527	0.0094	1.77E−08	–
rs11962411	6	15064540	***JARID2***	***JARID2***	t	g	0.8561	0.0495	0.0083	2.50E−09	+++
rs12879705	14	56017684	***KTN1***	***KTN1***	c	g	0.908	0.0535	0.0094	1.26E−08	+++
rs62101784	18	47216884	***LIPG***	***LIPG***	t	g	0.6312	0.0294	0.005	4.21E−09	+++
rs28827913	19	4132100	***MAP2K2***	***MAP2K2***	a	g	0.9125	0.0702	0.0121	6.03E−09	+++
rs16853076	3	168774754	***MECOM***	***MECOM***	t	c	0.9166	0.0479	0.0084	1.41E−08	+++
rs28649476	8	89113366	***MMP16***	***MMP16***	a	t	0.7211	−0.038	0.0067	1.43E−08	–
rs56108226	8	129224435	***MYC***	***MYC***	t	c	0.5848	−0.0364	0.0064	1.43E−08	–
rs1853196	9	14021167	***NFIB***	***NFIB***	t	c	0.7473	0.0386	0.0055	1.83E−12	+++
rs35643618	11	124614459	***NRGN***	***NRGN***	t	c	0.9163	−0.0567	0.0095	2.12E−09	–
rs9330355	4	138424568	***PCDH18***	***PCDH18***	a	c	0.8113	−0.0413	0.0063	4.02E−11	–
rs1019635	4	97047829	***PDHA2***	***PDHA2***	a	g	0.7688	0.0439	0.0074	2.81E−09	+++
rs145965565	9	98273305	***PTCH1***	***PTCH1***	t	g	0.8917	−0.0474	0.0085	2.90E−08	–
rs147216852	13	84391690	***SLITRK1***	***SLITRK1***	a	g	0.9699	−0.0943	0.0165	1.11E−08	–
rs62576842	9	85007039	***SPATA31D1***	***SPATA31D1***	a	c	0.966	−0.0915	0.0166	3.31E−08	–
rs35427	12	115556307	***TBX3***	***TBX3***	t	g	0.6217	0.0314	0.0052	2.03E−09	+++
rs112108303	1	218639669	***TGFB2***	***TGFB2***	a	g	0.9658	0.122	0.0206	3.09E−09	+++
rs140195071	7	65822883	***TPST1***	***TPST1***	t	c	0.9224	0.0658	0.012	3.79E−08	+++
rs879324	16	73068678	***ZFHX3***	***ZFHX3***	a	g	0.7913	0.0412	0.0057	5.98E−13	+++

**Previous variants**											

rs1565706	10	105458033	***SH3PXD2A***	***SH3PXD2A***	c	g	0.6176	0.035	0.005	2.32E−12	+++
rs16998073	4	81184341	***FGF5***	***FGF5***	a	t	0.6971	−0.0385	0.0052	1.49E−13	–
rs42036	7	92241451	***CDK6***	***CDK6***	c	g	0.7619	0.0504	0.0059	9.34E−18	+++
rs6847935	4	111696651	***PITX2***	***PITX2***	a	t	0.745	−0.0676	0.0056	4.62E−34	–
rs7091346	10	105608824	***SH3PXD2A***	***SH3PXD2A***	t	c	0.6207	−0.0447	0.0054	1.68E−16	–
rs7304841	12	20577593	***PDE3A***	***PDE3A***	a	c	0.5989	0.0424	0.005	1.42E−17	+++
rs7680240	4	111764972	***PITX2***	***PITX2***	a	c	0.5671	0.0379	0.0048	5.55E−15	+++

Findings were identified using fixed-effects inverse-variance weighted meta-analysis. The chromosomal position is based on GRCh37/hg19 reference. Genes with the same predictions as nearest gene and PoPs are shown. Allele1, effect allele; Allele2, non-effect allele; Eaf, effect allele frequency; StdErr, standard error.

### Screening of significant shared genes for *Cis*-eQTL and *Cis*-pQTL

**Figure 3:**
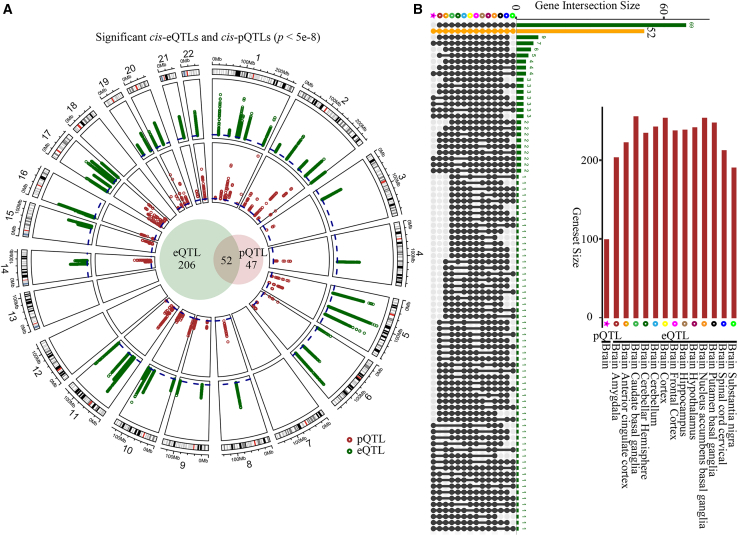
Significant *cis*-eQTLs, *cis*-pQTLs, and their overlap (A) Circular plot of the significant *cis*-eQTLs (green) and pQTLs (red) at *p* < 5 × 10^−8^ (blue dotted line). Considering only genes with both transcriptomics and proteomics measurements, we visualized the overlap of significant eQTLs and pQTLs in the circle center. (B) The overlapping characterization of eQTL (13 different brain site) and pQTL.

Given the importance of *cis*-regulatory genetic variants in influencing gene and protein expression, we analyzed *cis*-eQTL and *cis*-pQTL data to elucidate genotype-phenotype relationships underlying IS. Utilizing transcriptomic data from 13 distinct brain regions (GTEx v8) and proteomic data from the SYNAPSE resource, we examined a curated set of 258 genes with both transcriptomic and proteomic information (Table 2). We identified a substantial number of significant *cis*-eQTL SNP-gene pairs (*p* < 5 × 10^−8^), with the highest counts observed in the cerebral cortex (37,331 pairs) and cerebellum (35,987 pairs). In contrast, the *cis*-pQTL analysis yielded 2,390 significant SNP-protein associations (*p* < 5 × 10^−8^). Integration of these datasets revealed 69 genes consistently identified across the 13 brain-region-specific eQTL analyses, with 52 genes overlapping between the significant eQTL and pQTL sets (Figures 3A and 3B). These 52 shared genes were further analyzed across five key brain anatomical regions: cerebral cortex, cerebellum, basal ganglia, limbic system, and brainstem (Figure S2).^23^^,^^27^ Notably, for these 52 genes, a total of 1,408 significant SNP-protein pairs were identified in the pQTL analysis (Table S3).

**Table 2:** Summary of tested data and discovered *cis*-quantitative trait loci

	Tested				*p* < 0.05			*p* < 5 × 10^−8^		
	Parts	SNPs	Pairs	Genes	Pairs	Genes	Loci	Pairs	Genes	Loci
**pQTL**	Brain	26528	28253	258	9254	230	8934	2390	99	2373
**eQTL**	Brain_Amygdala	32386	33290	258	33290	258	32386	14226	203	14064
	Brain_Anterior_cingulate_cortex	41478	42731	258	42731	258	41478	22298	222	21706
	Brain_Caudate_basal_ganglia	57820	60865	258	60865	258	57820	33126	255	31978
	Brain_Cerebellar_Hemisphere	52259	54728	258	54728	258	52259	28878	234	28202
	Brain_Cerebellum	62720	66336	258	66336	258	62720	35987	242	34578
	Brain_Cortex	62001	65273	258	65273	258	62001	37331	253	36137
	Brain_Frontal_Cortex	48070	50013	258	50013	258	48070	27023	237	26534
	Brain_Hippocampus	41158	42971	258	42971	258	41158	20963	238	20539
	Brain_Hypothalamus	44086	45746	258	45746	258	44086	24390	241	24021
	Brain_Nucleus_accumbens_basal_ganglia	56035	58223	258	58223	258	56035	33200	253	31923
	Brain_Putamen_basal_ganglia	48886	51146	258	51146	258	48886	27713	247	27135
	Brain_Spinal_cord_cervical	32163	33785	258	33785	258	32163	16825	212	16506
	Brain_Substantia_nigra	27764	28502	258	28502	258	27764	13313	190	13160

Assessed *cis*-QTLs in human IS appendage tissue for mRNA and protein. Listed are the results for significance thresholds *p* < 0.05 and nominal *p* value < 5 × 10^−8^. Loci denote the number of independent loci derived by linkage disequilibrium clumping. eQTL, expression quantitative trait loci; pQTL, protein quantitative trait loci.

### Mendelian randomization with transcriptomics and proteomics identifies 7 genes for IS

To strengthen causal inference and identify robust candidate genes influencing IS, we performed two-sample MR analyses leveraging *cis*-genetic variants significantly associated with both transcript and protein abundance levels. Condition-specific *cis*-QTL variants (*p* < 5 × 10^−8^) identified from GTEx v8 transcriptomics and SYNAPSE proteomics data were utilized as instrumental variables, applied to European-ancestry GWAS meta-analysis results from the GWAS Catalog, MEGASTROKE, and Open GWAS cohorts.

To rigorously control for confounding and reduce bias from linkage disequilibrium (LD), we complemented MR analyses with summary-data-based Mendelian randomization (SMR) and Bayesian colocalization analyses. These integrative approaches further validated MR-derived causal inferences by ensuring alignment between genetic signals affecting expression levels and disease associations. Stringent thresholds (MR *p* < 0.05, SMR *p* < 0.05 with heterogeneity in dependent instruments (HEIDI) test *p* > 0.05, and colocalization posterior probability PP.H4 > 0.5) were applied to ensure robust causal gene identification.

**Figure 4:**
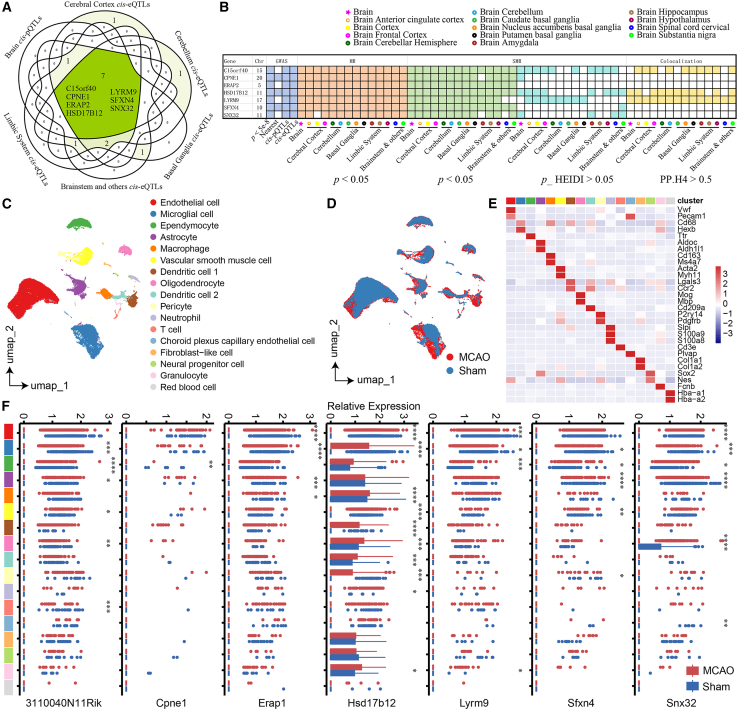
Complementary analysis results for the putative causal proteins (A) 7 candidate IS genes. (B) Prioritization of 7 candidate IS genes. (C) UMAP map showed 17 clusters from mouse IS models. (D) UMAP map showed distribution of MACO and sham after batch removal. (E) Heatmap showing the expression of known marker genes in these 17 clusters. (F) Comparison of 7 candidate IS genes expression between MACO and sham. *x* axis represents the average of normalized read counts per cell, *y* axis represents 17 cell type. *p* value was performed using the Wilcoxon test (∗: p ≤ 0.05, ∗∗: p ≤ 0.01, ∗∗∗: p ≤ 0.001, ∗∗∗∗: *p* < 0.0001). MACO, middle cerebral artery occlusion stroke mouse; sham, control mouse.

This comprehensive analytical strategy pinpointed seven candidate genes meeting all three analytical criteria (Figures 4A and 4B; Figure S3A; Table S4). Among these, three genes (*CPNE1*, *HSD17B12*, and *SFXN4*) were notably located within the novel genomic loci identified by our meta-analysis, highlighting their potential as high-priority candidates warranting future functional investigation and translational exploration in IS.

### scRNA-seq analysis identifies cell-type-specific expression of IS candidate genes

To delineate the cell-type-specific expression patterns of the 7 candidate IS genes, we conducted single-cell RNA sequencing (scRNA-seq) analyses using brain tissue samples from a murine middle cerebral artery occlusion (MCAO) stroke model. Three MCAO-treated mice and three sham-operated control mice were analyzed. After stringent quality control measures, our dataset included 58,363 high-quality cells expressing 18,017 genes. Principal-component analysis (PCA) and subsequent clustering analyses revealed 17 distinct cellular populations, which were confidently annotated based on the expression of well-established marker genes (Figures 4C–4E; Figure S3B). Notably, significant shifts in cell-type proportions were observed between MCAO and sham conditions (*p* < 0.05). Differential expression analysis highlighted significant alterations in three of the candidate genes *CPNE1*, *HSD17B12*, and *SFXN4* across distinct cell populations. Particularly striking was the significantly increased expression of *SFXN4* in endothelial cells within MCAO-treated mice, suggesting a potential role in endothelial dysfunction and associated pathophysiological changes in IS (Figure 4F).

### Mouse knockout models and druggability of IS candidate genes

To validate the biological significance of the candidate IS-associated genes identified in our integrative genetic analyses, we leveraged existing mouse knockout (KO) models from the Mouse Genome Informatics (MGI) database. Among the seven prioritized candidate genes, KO mouse models provided compelling evidence of functional roles for *HSD17B12*, *ERAP2*, and *SFXN4*. Specifically, deletion of *HSD17B12* was associated with disruptions in fatty acid metabolism, directly linking lipid metabolism dysregulation to cerebrovascular disease pathology. KO models for *ERAP2* and *SFXN4* indicated their involvement in immune regulation and iron metabolism, respectively, both processes implicated in stroke pathogenesis. Although KO models were unavailable for *C15orf40*, *CPNE1*, *LYRM9*, and *SNX32*, functional annotations suggest their potential roles in neuronal development, cell cycle regulation, and mitochondrial function, underscoring their plausible relevance to IS (Table 3). Furthermore, druggability analysis revealed that three candidate genes (*CPNE1*, *HSD17B12*, and *ERAP2*) encode proteins that are currently targeted by approved or clinically investigated drugs, highlighting their translational potential for IS therapy development (Table 3).^16^^,^^17^^,^^24^

**Table 3:** Summary of druggability and KO mouse models

Genes	Druggability classification	Generic name	MGI mouse KO models
C15orf40	non-druggable		no
CPNE1	approved (small molecule)	theophylline	no
ERAP2	not approved (small molecule)	scopoletin	yes
	not approved (inhibitor)	COMPOUND 2G	
	not approved (inhibitor)	COMPOUND 17	
	investigational (small molecule)	tosedostat	
	investigational (small molecule)	esculetin	
	not approved (inhibitor)	Compound 3v	
HSD17B12	investigative (small molecular drug)		yes
LYRM9	non-druggable		no
SFXN4	non-druggable		yes
SNX32	non-druggable		no

Mouse knockout models queried from MGI. Druggability queried from DrugBank, UniChem, TTD, and DGIdb. MGI, Mouse Genome Informatics. KO, knock out.

### Developing a knowledge base and intelligent assistant for stroke genetics

**Figure 5:**
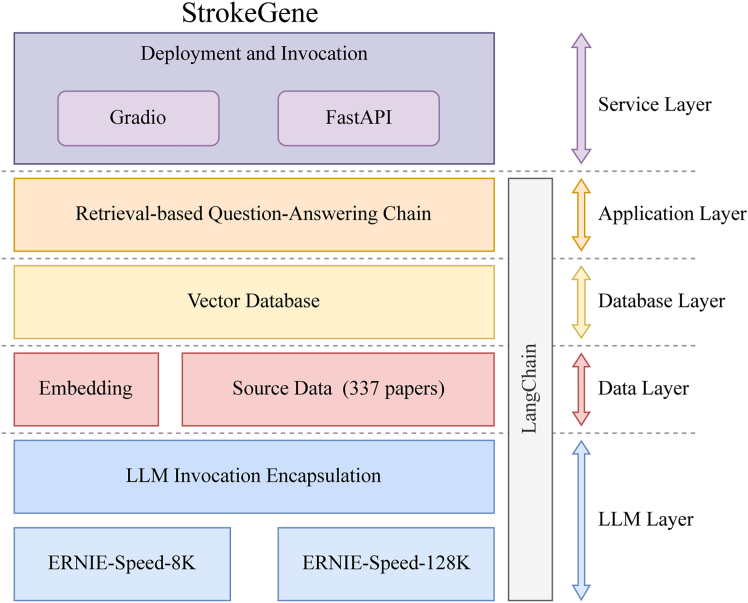
The architecture of StrokeGene

Given the extensive body of genetic research on IS, encompassing numerous susceptibility SNPs located in diverse genomic regions, we curated a comprehensive knowledge base from 337 genetic association studies specifically focusing on stroke susceptibility loci. Leveraging advancements in LLMs, we developed StrokeGene, an intelligent assistant designed to facilitate rapid querying, analysis, and interpretation of stroke-related genetic literature, thereby streamlining and enhancing the research workflow (Figure 5).

StrokeGene is built upon state-of-the-art natural language processing (NLP) models-specifically ERNIE-Speed-8K and ERNIE-Speed-128K-allowing efficient and precise extraction of information from unstructured literature data. To convert raw research publications into a structured knowledge repository, original PDF documents were initially transformed into Markdown format using the MinerU extraction tool. Subsequently, textual data were embedded using the M3E word embedding model, effectively capturing semantic relationships through optimized vector representations, thus supporting robust downstream tasks, including intelligent question-answering and literature-driven reasoning.

The intelligent querying mechanism in StrokeGene operates via a sophisticated question-answer chain (QA chain) process, wherein user-submitted queries are parsed, contextually enriched using relevant knowledge-base entries, and processed by underlying NLP models to generate accurate and context-aware responses. For optimal performance and user experience, StrokeGene’s backend was developed using the lightweight Flask framework, ensuring rapid request processing and efficient resource management. Meanwhile, the frontend user interface was constructed with the Bootstrap framework, providing a responsive, intuitive, and user-friendly platform. StrokeGene is freely accessible to the research community at https://github.com/QIANJINYDX/StrokeGene, promoting widespread utilization and collaborative advancement in stroke genetics research.

## Discussion

In this study, we conducted an extensive genome-wide meta-analysis leveraging three major IS GWAS datasets, the GWAS Catalog, MEGASTROKE, and Open GWAS, to identify 124 novel genetic loci associated with IS, alongside confirmation of 33 previously recognized loci. The newly discovered loci significantly advance our understanding of the genetic architecture underlying IS, with marked enrichment observed within genomic regions implicated in cerebrovascular regulation, inflammation, and metabolic control. These findings not only elucidate critical biological mechanisms underpinning stroke pathophysiology but also reveal potential translational opportunities. Specifically, the identification of loci involved in vascular dysfunction and inflammatory pathways suggests novel targets for pharmacological intervention, potentially guiding precision medicine strategies tailored to patients’ individual genetic profiles, thereby enhancing preventive and therapeutic outcomes.

Through detailed gene annotation and integrative functional analyses, we prioritized several promising candidate genes, including *CPNE1*, *HSD17B12*, and *SFXN4*, that appear to play pivotal roles in IS pathogenesis. These genes are robustly implicated in diverse biological pathways such as lipid metabolism, immune modulation, and iron homeostasis, suggesting multifaceted mechanisms by which genetic predisposition contributes to IS risk and progression.^28^^,^^29^^,^^30^^,^^31^ Clinically, these insights are particularly significant, offering potential biomarkers for early detection, patient stratification, and personalized risk assessment. For example, the involvement of *HSD17B12* in lipid regulation may facilitate the identification of dyslipidemia-associated stroke risk, potentially enabling early intervention strategies even before clinical manifestations emerge. The integration of eQTLs and pQTLs further strengthens the functional relevance of these candidate genes across multiple brain regions and cell types, thereby enhancing their clinical translation potential for therapeutic targeting.^32^

To further elucidate the potential mechanisms by which our candidate genes may influence stroke risk, we examined their functional annotations and relevant literature. *CPNE1* is a calcium dependent phospholipid binding protein involved in cellular signaling and membrane dynamics, with emerging evidence linking it to lipid metabolism and vascular smooth muscle cell function both crucial in atherosclerotic plaque formation.^33^^,^^34^ *SFXN4*, encoding an inner mitochondrial membrane protein, is essential for mitochondrial respiration and erythropoiesis, and studies have suggested that erythropoiesis stimulating agents may inadvertently increase stroke risk through hyperviscosity related mechanisms.^35^^,^^36^^,^^37^ *ERAP2* contributes to antigen processing and has been implicated in immune-related conditions such as preeclampsia and hypertension, both of which are stroke risk factors; it may modulate stroke susceptibility through immunoinflammatory pathways.^38^^,^^39^^,^^40^^,^^41^^,^^42^ *HSD17B12*, primarily expressed in platelets, is key in fatty acid metabolism and lipid biosynthesis; its dysregulation may promote atherogenesis and platelet-mediated thromboembolism in cerebral ischemia.^43^^,^^44^^,^^45^ While *C15orf40* remains poorly characterized, its mitochondrial association suggests a possible link to cellular energetics. Similarly, *LYRM9* may participate in oxidative phosphorylation, and mitochondrial dysfunction is a known contributor to neurovascular injury.^46^^,^^47^^,^^48^ *SNX32* is involved in endosomal trafficking and receptor recycling, and its dysregulation can influence inflammatory signaling and vascular integrity, both relevant to stroke pathogenesis.^49^^,^^50^^,^^51^ Together, these mechanistic insights support the biological plausibility of our candidate genes and align with key pathways implicated in stroke, including lipid metabolism, immune regulation, mitochondrial function, and vascular homeostasis. These findings underscore the need for further experimental validation to elucidate causal mechanisms and therapeutic implications.

A critical strength of this investigation is our use of a multilayered, integrative approach combining multiple large-scale genomic datasets with transcriptomic and proteomic data.^17^^,^^52^^,^^53^^,^^54^ This comprehensive multi-omics framework overcomes the inherent limitations of single-dimensional analyses, significantly improving the statistical power and biological interpretability of identified genetic associations. From a translational perspective, this integration paves the way for developing composite biomarkers that simultaneously incorporate genomic, transcriptomic, and proteomic information, thus providing a robust basis for improved predictive modeling of IS risk. Additionally, our rigorous application of MR, SMR, and colocalization analyses notably reduces the potential for confounding and linkage disequilibrium biases, enhancing the reliability and reproducibility of our identified causal loci.

Recognizing the importance of rapidly translating these genetic insights into actionable knowledge, we developed StrokeGene, an intelligent assistant built upon advanced LLMs and NLP techniques. StrokeGene systematically integrates a wealth of genetic research literature related to IS, enabling efficient querying and analytical interpretation of complex genetic associations. By leveraging cutting-edge NLP technologies, this tool assists researchers in swiftly extracting relevant information regarding genetic loci, elucidating potential gene-environment interactions, and identifying promising avenues for functional validation. As stroke genetics research evolves, StrokeGene will serve as an indispensable resource, accelerating both basic research and translational discoveries that could significantly impact clinical practice.

Despite these substantial advancements, several limitations should be acknowledged. Although our meta-analysis incorporated data from large-scale, predominantly European-ancestry GWAS cohorts, the generalizability of identified loci across diverse ethnic populations remains uncertain and necessitates further validation studies. Additionally, GWAS inherently identifies genomic variants statistically associated with disease susceptibility, but does not directly establish causality or functional effects. Consequently, the precise molecular consequences of these variants particularly their impact on gene expression and protein function require rigorous validation through experimental methods such as cell-based assays and animal models. Moreover, while we effectively integrated transcriptomic and proteomic datasets, these associations primarily reflect statistical correlations rather than direct biological causation, highlighting the importance of subsequent experimental confirmation to elucidate the mechanistic roles of these candidate genes in IS pathology.

Looking forward, this study provides novel, clinically actionable insights into the genetic underpinnings of IS, highlighting potential molecular targets that merit focused translational and therapeutic investigation. Future research should prioritize functional validation of these candidate genes using *in vivo* animal models and human-derived cellular assays to dissect their precise roles in stroke pathogenesis. As the accumulation of multi-omics datasets continues to expand, the integration of epigenomic and metabolomic information could further refine our understanding of the complex molecular networks implicated in stroke. Ultimately, combining genetic, epigenetic, and metabolic profiles could enable clinicians to implement precision-medicine approaches targeting multiple pathogenic pathways simultaneously, significantly improving preventive strategies, therapeutic interventions, and clinical outcomes for IS patients.

### Conclusion

This study identified 124 novel genetic loci associated with IS through a comprehensive meta-analysis of large-scale GWAS datasets. Several candidate genes particularly *CPNE1*, *HSD17B12*, and *SFXN4* were highlighted, implicating critical pathways such as lipid metabolism, immune regulation, and iron homeostasis. Integrative multi-omics analyses, including eQTL, pQTL, and MR, supported their potential causal roles in IS pathogenesis, further validated by scRNA-seq and mouse KO data. Additionally, the development of StrokeGene, an intelligent knowledge base powered by LLMs, provides researchers with a powerful tool for rapidly exploring genetic mechanisms underlying stroke. These findings lay essential groundwork for precision medicine approaches aimed at enhancing stroke prevention and personalized treatment strategies.

## Materials and methods

### GWAS datasets

Our study integrated GWAS data from a substantial total of 2,396,043 participants, derived from three major publicly available GWAS resources: GWAS Catalog (GCST90104540; cases: 62,100; controls: 1,234,808),^25^ MEGASTROKE (cases: 34,593; controls: 624,214),^18^ and Open GWAS (ebi-a-GCST006908; cases: 34,217; controls: 406,111).^26^ To minimize population stratification bias, all GWAS summary statistics were derived from studies conducted in populations of European ancestry. This choice was made to minimize population stratification bias and ensure genetic homogeneity across exposure and outcome datasets. Furthermore, to reduce the risk of bias due to sample overlap which can potentially inflate causal estimates in two-sample MR, we carefully selected non-overlapping GWAS datasets where possible. When complete exclusion could not be confirmed due to lack of individual level data, we ensured that the proportion of potential sample overlap was minimal and unlikely to materially affect the MR estimates. GWAS summary statistics, including effect sizes, *p*-values, and standard errors, were directly retrieved from the respective source datasets. All genomic variant positions were consistently mapped to the GRCh37 (hg19) reference genome to facilitate accurate integration and comparability across studies. The analysis involving the X chromosome was restricted specifically to the meta-analyzed data to address the unique inheritance patterns and analytical complexities associated with sex chromosomes, thereby ensuring methodological rigor and robustness in our variant association analyses.

### GTEx and SYNAPSE QTL datasets

To investigate the functional impacts of genetic variants on gene and protein expression in IS, we employed *cis*-eQTL data from GTEx (Genotype-Tissue Expression) version 8, which includes expression data across 13 distinct human brain regions. Additionally, we utilized *cis*-pQTL data available through the SYNAPSE repository.^55^ Summary statistics for variant-gene pairs were downloaded from the GTEx portal: https://www.gtexportal.org/home/downloads/adult-gtex/qtl, whereas variant-protein associations were obtained from SYNAPSE: https://www.synapse.org/Synapse:syn23627957. The GTEx dataset utilized the GRCh38 (hg38) reference assembly, while the SYNAPSE dataset was annotated using GRCh37 (hg19). To ensure accurate cross-dataset integration, comprehensive SNP annotation data for both genome builds were also obtained from the GTEx resource.

We selected 13 brain regions from the GTEx dataset, including the anterior cingulate cortex, frontal cortex, cortex, caudate basal ganglia, putamen basal ganglia, nucleus accumbens basal ganglia, amygdala, hippocampus, hypothalamus, cerebellum, cerebellar hemisphere, substantia nigra, and spinal cord cervical. Several of these regions, particularly the anterior cingulate cortex, frontal cortex, cortex, caudate, putamen, and nucleus accumbens, are anatomically located within or closely associated with the middle cerebral artery (MCA) territory, which is the most frequently affected area in IS.^56^^,^^57^ These regions include parts of the lateral frontal, parietal, and temporal lobes, as well as deep subcortical nuclei. Other regions, such as the amygdala, cerebellum, hippocampus, and hypothalamus, while not typically located within the MCA territory, were included to allow comprehensive analysis of transcriptomic profiles across diverse brain regions and to account for possible indirect or secondary effects of stroke on the central nervous system.

### Meta-analysis and SNP gene annotation

To systematically uncover genetic loci associated with IS, we conducted a fixed-effects inverse-variance weighted meta-analysis across three independent GWAS datasets MEGASTROKE (*n* = 658,807), GWAS Catalog (*n* = 1,296,908), and Open GWAS (*n* = 440,328) using METAL software (version 2011-03-25).^58^ This integrative analysis resulted in 24,640,797 genetic association signals. Novel loci were stringently defined as variants achieving genome-wide significance (*p* < 5 × 10^−8^) and located at least 500 kb away from previously identified indexed SNPs. For gene annotation of these significant loci, we implemented a dual annotation strategy: (1) prioritizing the nearest gene within ±500 kb of each indexed SNP, based on the assumption that genomic proximity indicates higher functional relevance; and (2) employing the PoPSmethod,^59^ a sophisticated multi-dimensional scoring approach leveraging GWAS summary statistics, expression data, pathway information, and predicted protein-protein interactions to further refine gene prioritization.

Specifically, we annotated all candidate genes located within a ±500 kb genomic window surrounding each indexed SNP using the GENCODE hg19 annotation. The closest gene to each SNP was initially prioritized, based on spatial proximity. Subsequently, PoPS analysis was applied to these annotated genes, allowing for a comprehensive, unbiased prioritization of genes based on broader genomic context and biological plausibility.

### Functional enrichment analysis

To elucidate the biological processes and pathways implicated by the identified IS-associated genetic loci, we performed comprehensive functional enrichment analyses. Genes prioritized by both genomic proximity and PoPS methodology were consolidated into a unified candidate gene set. This gene set was subsequently submitted for enrichment analysis using Metascape,^60^ a robust bioinformatics resource integrating multiple ontologies and pathway databases. Specifically, our analyses included Gene Ontology (GO), Kyoto Encyclopedia of Genes and Genomes (KEGG), and Reactome databases.^61^ Pathways achieving statistical significance at a false discovery rate (FDR) threshold of less than 0.05 were identified as significantly enriched, providing critical insights into potential molecular mechanisms underlying IS pathophysiology.

### Mendelian randomization, SMR, and colocalization analyses for *cis*-e/pQTL data

To systematically investigate the causal relationship between genetic variants influencing gene and protein expression (e/pQTLs) and IS risk, we performed a series of integrative genetic analyses, including two-sample MR, SMR, and colocalization analyses. Initially, approximate conditional analyses were conducted to detect secondary genetic signals within identified genomic regions, thereby ensuring the comprehensive capture of independent variant associations.^62^ Instrumental variables for MR analyses were rigorously selected from *cis*-eQTL and *cis*-pQTL datasets (GTEx and SYNAPSE) by imposing a strict genome-wide significance threshold (*p* < 5 × 10^−8^) and limiting the *cis*-region definition to ±1 Mb around protein-coding genes. Ultimately, 52 genes demonstrated overlapping significant *cis*-eQTL and *cis*-pQTL signals and were subsequently prioritized for causal inference analyses.

Two-sample MR analyses were conducted using the TwoSampleMR package (version 0.5.6; https://mrcieu.github.io/TwoSampleMR/) in R.^59^ Specifically, the Wald Ratio method was employed for single-variant instrumental variables, whereas the inverse-variance weighted (IVW) MR approach was utilized for analyses involving multiple instrumental variants. Variant-level heterogeneity among MR estimates was statistically assessed using Cochran’s Q test within the TwoSampleMR framework. Furthermore, scatterplots comparing variant effects on gene/protein expression versus IS were generated to visually validate instrumental variable consistency. Statistically significant MR results were defined by *p* < 0.05. Potential directional pleiotropy was evaluated using the MR-Egger intercept test, with intercept estimates, standard errors, and *p*-values reported for genes represented by three or more instrumental variants. A non-zero MR-Egger intercept would indicate potential bias due to pleiotropy.^63^

To further confirm the robustness of causal associations identified through MR, we implemented SMR analyses using *cis*-e/pQTL summary statistics as instrumental variables.^64^ Genes passing the significance threshold (SMR *p*-value <0.05) underwent further evaluation through the HEIDI test (p_HEIDI >0.05), distinguishing genuine pleiotropy from linkage disequilibrium (LD)-driven associations. Bayesian colocalization analysis, conducted via the COLOC R package,^65^ provided additional validation by assessing the probability that e/pQTL signals and IS GWAS signals shared a single causal variant. Strong evidence of colocalization was designated by a posterior probability of hypothesis 4 (PP.H4) > 0.5, indicative of a shared causal variant. Conditional analyses of e/pQTL data identified distinct association signals, which were subsequently incorporated into the colocalization framework alongside marginal (uncorrected) results, thereby enhancing analytical rigor. Lastly, we explored overlaps between significantly identified MR genes and established Gene Ontology (GO) and KEGG pathways to further validate biological relevance.

We considered a posterior probability of colocalization (PP.H4) > 0.5 as evidence of a shared causal variant between gene expression and IS. This threshold is commonly used in colocalization studies and represents a balance between false positive and false negative rates in the context of complex traits.^65^^,^^66^ While more stringent thresholds (e.g., PP.H4 > 0.8) may increase specificity, they may also reduce sensitivity, especially when tissue-matched eQTL sample sizes are modest.

### scRNA-seq data acquisition and processing

To explore the gene expression profiles of IS in an *in vivo* model, we downloaded (scRNA-seq data from IS mouse models (GEO: GSE174574)^67^ available on the GEO database. The dataset includes brain tissue samples from mice subjected to MCAO and corresponding control subjects. Data integration was performed using the “Seurat” R package,^68^ with stringent filtering applied to exclude low-quality cells. Cells with fewer than 200 detected genes or those expressing genes found in fewer than three cells were excluded. Normalization of gene expression was carried out using linear regression, and the top 2000 most variable genes were selected for further analysis. PCA was applied to reduce dimensionality, with the top 30 components retained for further analysis. Uniform Manifold Approximation and Projection (UMAP) was used for further dimensionality reduction and visualization of the data.^69^^,^^70^^,^^71^ Cell type clusters were annotated manually based on established marker genes.^72^

### Mouse model phenotype query and druggability assessment

To evaluate the functional implications of prioritized candidate genes in IS pathology, we queried the MGI database (http://www.informatics.jax.org/) for available KO mouse models exhibiting phenotypes relevant to IS.^73^ MGI employs standardized nomenclature and controlled ontologies, such as the Mammalian Phenotype Ontology, Gene Ontology, and Mouse Developmental Anatomy Ontology, ensuring accurate phenotype annotations. Phenotypic abnormalities related to IS were manually curated and systematically documented.

To identify candidate gene products amenable to pharmacological intervention, druggability assessments were conducted using comprehensive databases, including DrugBank.^74^ UniChem,^75^ TTD,^76^ and DGIdb.^77^ Approved drug indications were carefully reviewed to discern whether drug targets were implicated in disease states beyond IS, thus preemptively addressing potential biases introduced during MR analyses.

## Data availability

StrokeGene is freely available at https://github.com/QIANJINYDX/StrokeGene.

## Acknowledgments

This work was supported by the 10.13039/501100001809National Natural Science Foundation of China (82160244). The MEGASTROKE project received funding from sources specified at http://www.megastroke.org/acknowledgments.html.

## Author contributions

M.W.: conceptualization, methodology, validation, and writing – original draft. C.D.: methodology, validation, and writing – original draft. X.D.: formal analysis and validation. T.Z.: formal analysis and validation. X.Y.: formal analysis. F.D.: supervision and project administration. Guangyan Wang: investigation and formal analysis. Y.Z.: writing – review and editing and supervision. H.C.: writing – review and editing, supervision, and project administration. Guangming Wang: conceptualization, writing – review and editing, supervision, project administration, and funding acquisition.

## Declaration of interests

The authors declare no competing interests.

## Declaration of generative AI and AI-assisted technologies in the writing process

During the preparation of this work, the authors used Kimi in order to improve language and readability. After using this service, the authors reviewed and edited the content as needed and took full responsibility for the content of the publication.

## Supplemental information

Document S1. Figures S1–S3

Table S1. Loci reported for IS in the meta-analysis

Table S2. KEGG and GO enrichment result

Table S3. *cis*-QTL of 52 genes from brain

Table S4. MR_SMR_Colocalization result

Document S2. Article plus supplemental information
