# Supplementary material for: Multi-omics integrative analysis reveals novel genetic loci and candidate genes for ischemic stroke
Source: Mol Ther Nucleic Acids. 2025 Jul 17;36(3):102633. doi: 10.1016/j.omtn.2025.102633 (PMC12328896; doi:10.1016/j.omtn.2025.102633)
Supplement: Document S1. Figures S1–S3 [file mmc1.pdf]

OMTN, Volume *36*

Supplemental information

Multi-omics integrative analysis reveals novel genetic loci and candidate genes for ischemic stroke

Min Wang, Chong Xu, Xiaoshan Du, Tian Zhu, Xitong Yang, Fuhui Duan, Guangyan Wang, Yongchun Zuo, Huaqiu Chen, and Guangming Wang

Fig. S1

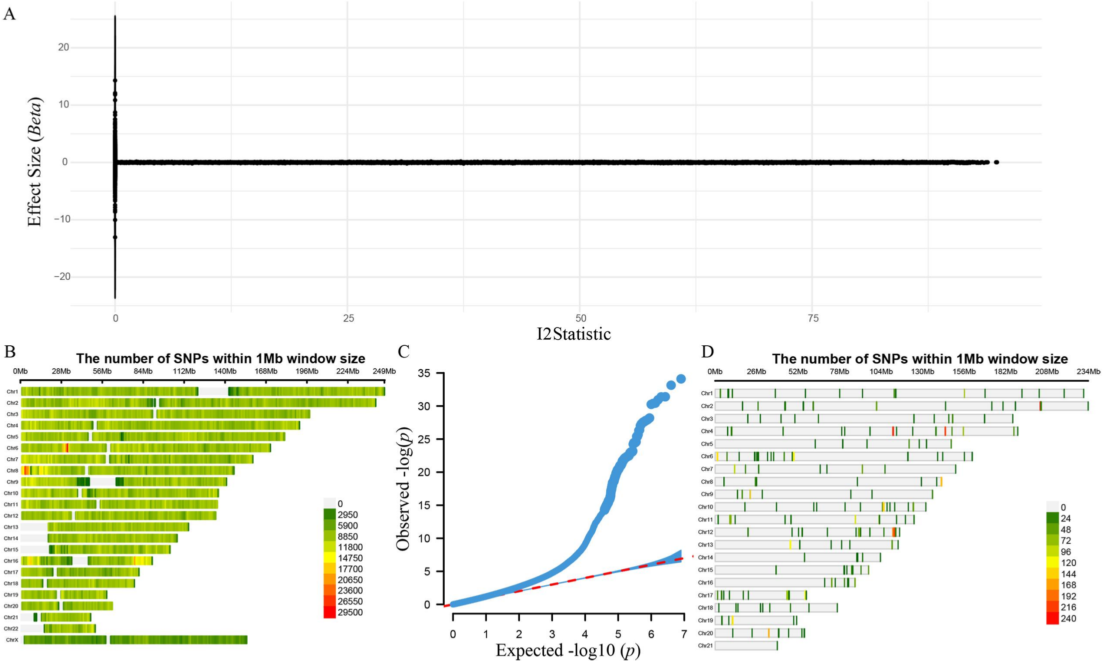

Fig. S1 Meta-analysis of three IS GWAS datasets. A Scatter plot shows the relationship between the I^2 heterogeneity statistics and the effect size of various genetic variants (SNPs). The x-axis represents the I^2 statistic, while the y-axis shows the estimated effect sizes (beta coefficients) of the SNPs on the outcome. Vertical error bars indicate the 95% confidence intervals for the effect estimates. B Genome density map shows the distribution of all SNP of three IS GWAS datasets density in different chromosomes in the genome. The color change from dark green (low density) to red (high density) reveals different distribution patterns of SNPS on chromosomes. C The Q-Q plot presents the observed p-value distribution compared to the expected distribution. Most of the points were close to the diagonal, indicating no significant deviation. However, points that deviate significantly suggest the presence of potential true positive signals or false positive results. D Genome density map shows the distribution of significant SNP of three IS GWAS datasets density in different chromosomes in the genome after meta-analysis. The color change from dark green (low density) to red (high density) reveals different distribution patterns of SNPS on chromosomes.

Fig. S2

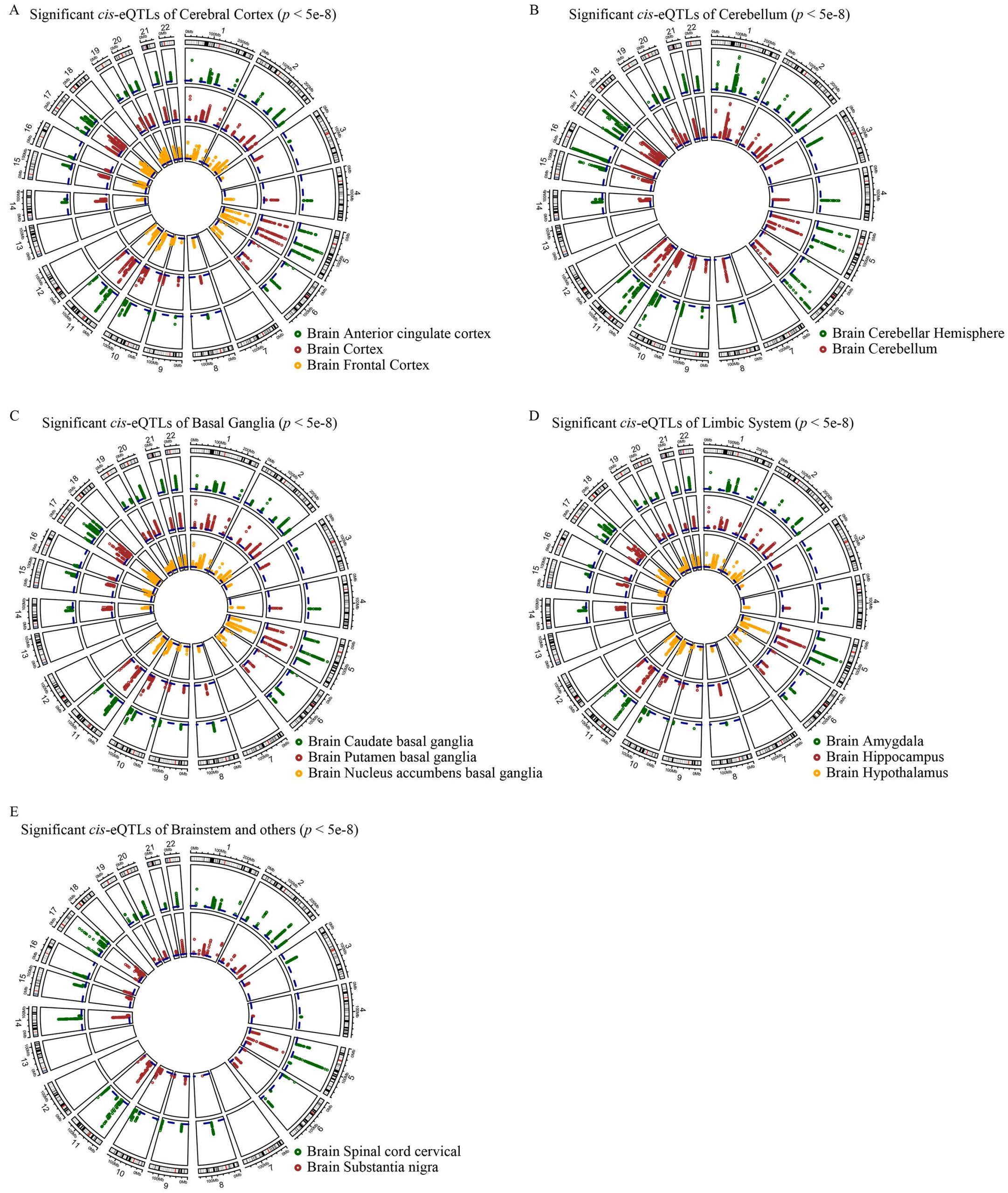

Fig. S2 The significant cis-eQTLs of 5 anatomical Regions of the Brain. Circular plot of the significant cis-eQTLs at $p < 5\times10^{-8}$ (blue dotted line) from cerebral cortex (A), cerebellum (B), basal ganglia (C), limbic system (D), brainstem and others (E). Considering only genes with both transcriptomics and proteomics measurements.

Fig. S3

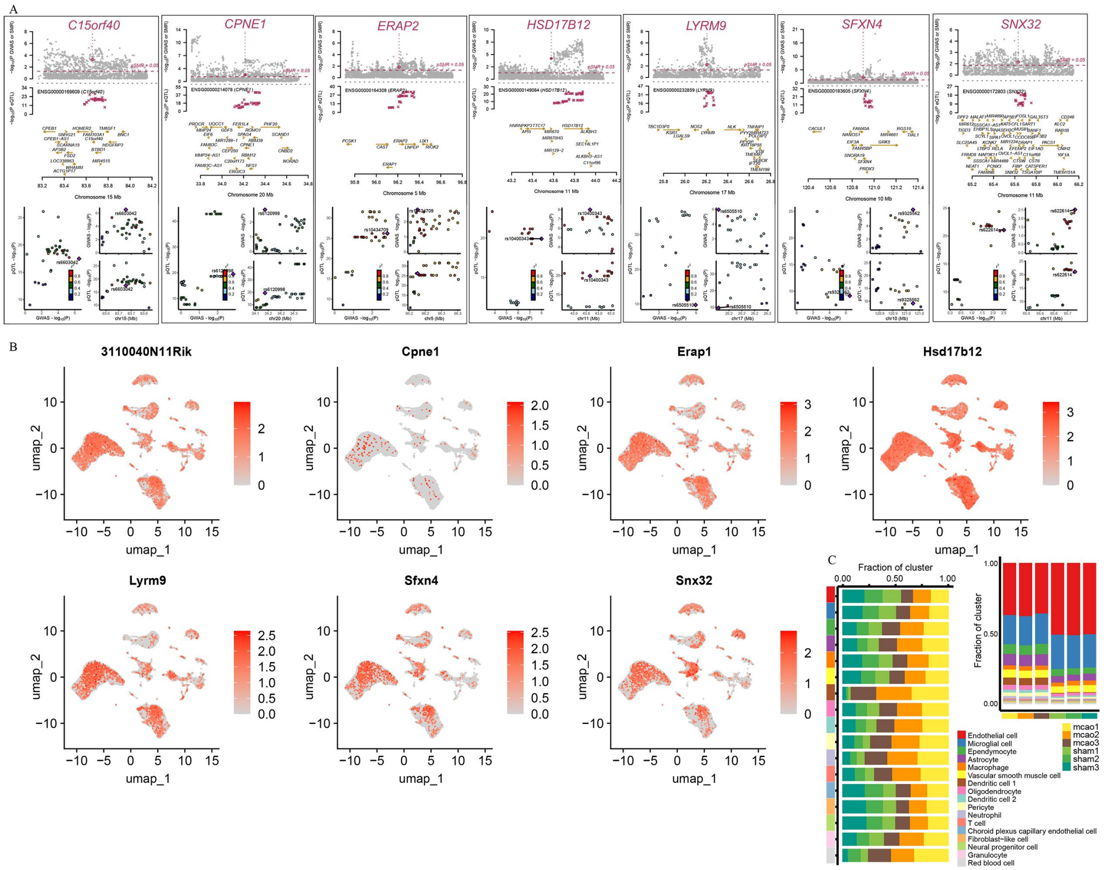

Fig. S3 Character of 7 candidate IS genes. A SMR and colocalization analyses prioritized IS causal genes. The top panels indicate the SMR result between eQTL and GWAS (all SMR FDR < 0.05; HEIDI test P > 0.05), while the bottom panels show the locus comparisons between GWAS and pQTLs by colocalization analysis (all PPH4 > 0.5). The r^2 value indicates the linkage disequilibrium (LD) between the variants and the top SNPs. B UMAP map shows expression of 7 candidate IS genes.

Table S1. Loci reported for IS in the meta-analysis
Table S2. KEGG and GO enrichment result
Table S3. cis-QTL of 52 genes from brain
Table S4. MR_SMR_Colocalization result
